# Associations between sexual identity, living with disability, bully victimisation, and HIV status and intimate partner violence among residents in Nigeria

**DOI:** 10.1186/s12889-022-14186-6

**Published:** 2022-09-16

**Authors:** Morenike Oluwatoyin Folayan, Ibidunni Olapeju Oloniniyi, Ikenna Nwakamma, Erva-Jean Stevens-Murphy, Gabriel Undelikwo, Joanne Lusher

**Affiliations:** 1 Department of Child Dental Health, Obafemi Awolowo University, Ile-Ife, Nigeria; 2 Department of Mental Health, Obafemi Awolowo University, Abuja, Nigeria; 3 Coalition of Civil Society Networks On HIV and AIDS, Abuja, Nigeria; 4 UNAIDS, Abuja, Nigeria; 5 Regent’s University London United Kingdom, London, England

**Keywords:** Sexual minority, Heterosexuals, Emotional violence, Physical violence, Sexual violence, Nigeria

## Abstract

**Background:**

The aim of the study was to determine the associations between sexual identity, disability and HIV status and bullying victimisation, and a history of physical, emotional and sexual violence in Nigeria.

**Methods:**

This was a secondary analysis of a primary dataset generated through an online survey conducted between February 7 and 19, 2021. The 3197 participants for the primary study were recruited through snowballing. The dependent variables were physical, emotional and sexual violence. The independent variables were sexual identity (heterosexual and sexual minority), HIV status (negative, positive and unknown), bullying victimisation (yes/no) and living with disability (yes/no). A multivariate logistic regression model was developed for each form of IPV. Each model was adjusted for age, sex assigned at birth, marital status and education level.

**Results:**

Respondents living with HIV had higher odds for physical (AOR: 2.01; 95% CI: 1.46–2.76; *p* < 0.001), sexual (AOR: 2.17; 95%CI: 1.55–3.05; *p *< 0.001), and emotional (AOR: 1.59; 95%CI: 1.24–2.06; *p* < 0.001) violence. Also, those with history of bullying victimisation had higher odds for physical (AOR: 3.79; 95%CI: 2.86 – 5.68; *p* < 0.001), sexual (AOR: 3.05; 95%CI: 2.27 – 4.10; *p* < 0.001) and emotional (AOR: 2.66; 95%CI: 2.10 – 3.37; *p* < 0.001) violence. In addition, females had higher odds of physical (AOR: 1.52; 95%CI: 1.13–2.043; *p* < 0.001) and sexual (AOR: 1.83; 95%CI: 1.34 – 2.50; *p* < 0.001) violence; and respondents cohabiting (AOR: 1.95; 95%CI: 1.12 – 3.28; *p* = 0.012) had higher odds for emotional violence. Respondents who were married have significantly lower odds of experiencing physical (AOR: 0.66; 95%CI: 0.45 – 9.60; *p* = 0.029), sexual (AOR: 0.40; 95%CI: 0.26 – 0.62; *p* < 0.001) and emotional (AOR: 0.68; 95%CI: 0.50 – 0.93; *p* = 0.015) violence when compared to singles. Younger respondents also had lower odds of experiencing sexual violence (AOR: 0.97; 95%CI: 0.95–0.99; *p* = 0.016).

**Conclusion:**

HIV positive status and bullying victimisation seem to increase the risk for all forms of IPV while the experience of IPV did not differ by sexual identity and disability status. The associations between age, sex, marital status and IPV may suggest moderating roles of the factors taking cognisance of the cultural context of these relationships. Future relational analysis is necessary to further understand the pathways for the associations found between the variables in this study.

**Supplementary Information:**

The online version contains supplementary material available at 10.1186/s12889-022-14186-6.

## Introduction

Intimate partner violence (IPV) describes a pattern of physical (slapping, punching, shoving, or otherwise physically hurting on purpose), sexual (physical coercion into sexual intercourse or demeaning sexual acts), emotional (belittling, humiliating, intimidating, and threatening to harm a person or someone they care about), and psychological (verbal abuse, threats, withholding allowance, fear of spouse, and refusal of food for a person) violence that occurs between individuals who have a current or former dating, marital or cohabiting same-sex or opposite sex relationship [1]. IPV is used to gain or maintain power and control over an intimate partner [2]. This is usually perpetrated by men towards women, although it can occur in either direction [3]. It is a strategy for men to resolve a crisis of male identity caused by multiple factors many of which are context specific [4]. IPV negatively affect women’s physical, mental, sexual, and reproductive health, and may increase the risk of contracting HIV in some settings [5].

An estimated 27% of women aged 15–49 years worldwide have ever experienced either physical and/or sexual IPV or non-partner sexual violence since the age of 15 years and 13% had experienced IPV in the last one year [5]. This lifetime prevalence rates range from 13 to 61% for physical IPV, from 6 to 59% for sexual IPV [6]. The lifetime prevalence of physical and/or sexual IPV or non-partner sexual violence in Africa is 33% and 20% in the last one year [5]. This is higher than the lifetime prevalence of 22% and 6% in the last one-year estimates for high income countries [5]. The lifetime prevalence rates for emotional IPV ranges from 20 to 75% [6].

The prevalence of IPV is also high in patriarchal societies like Nigeria where community norms justify IPV against women [7], and there is cultural acceptance of wife-beating among men and women, and the right of the male discipline or control female behaviour [8–10]. Also, women’s engagement in cash work was positively associated with physical and sexual IPV victimisation [9]. The lifetime prevalence of physical and/or sexual IPV or non-partner sexual violence in Nigeria is 24% and 13% in the last one year [5]. Reports from the Nigerian national population commission estimated that women’s lifetime exposure to IPV from their current husband or partner is 19% for emotional violence, 14% for physical violence, and 5% for sexual violence [11]. The experience of IPV is moderated by age in Nigeria; it increases, peaks, and then declines with age [11]. Similarly, educational status also moderates the experience of IPV as the experience of IPV decreases as education status increases [11]. The protective effect of education is, however, lost in a society that justifies wife-beating as a woman’s non-approval of IPV may not be enough to reduce her risk of IPV [11].

The global prevalence of IPV is higher among people living with HIV. It is estimated that 39% of women living with HIV had experienced at least one type of IPV, with prevalence ranging from 26% with a history of physical IPV, 17% with a history of sexual IPV, 28% with a history of emotional IPV, and 23% with a history of psychological IPV [12]. Women living with HIV who disclose their HIV status are more likely to experience IPV than HIV negative women [13]. In Kano, Nigeria, a fifth of women living with HIV experienced domestic violence following HIV diagnosis with 59.3% of them experiencing emotional violence, 30.0% experiencing physical violence and 10.7% experiencing sexual violence [14]. The risk for IPV was higher for younger women, those who were divorced/widows, those with lower education status and those who disclosed their HIV status [14].

Sexual minority individuals (lesbians, gays and bisexuals) also experience IPV [15] at equal to or greater rates than that observed among heterosexuals [16]. Risk factors for IPV among sexual minority individuals are comparable to those documented among heterosexual individuals. However, internalized homonegativity is an additional risk factor for IPV among sexual minorities; and this may explain the higher rates of IPV among sexual minorities [16]. The level of minority stress is high for sexual minorities in Nigeria because of the unsupportive political, culture, religious and legal environment [17–19].

Just as gender, HIV status and sexual identity increases the risk for IPV, so also does disability status: women living with a disability are more likely to experience IPV than their able-bodied counterparts [20]. The prevalence of sexual IPV among women with disabilities ranged from 13.5% to 17.1% and 36.0% experience physical violence in New Zealand [ref]. The one-year prevalence estimates were 0.6% for rape, 2.3% for sexual violence other than rape, 4.0% for physical violence and 13.9% for psychological violence in the US. This prevalence was 2.5% for sexual violence other than rape, 4.7% for physical violence, and 18.1% for psychological violence [20]. The prevalence of IPV among people with disability was 1.18% with a non-significant increased odds when compared with those without disability in Australia [21]; and 3.8% of those with physical disabilities and 6.7% of those with mental disabilities experiencing IPV in Denmark in the past year [22]. Women with disability are at risk of IPV because of the potential for physical dependence on an intimate partner, higher levels of poverty, social isolation, and perceived vulnerability by perpetrators [23]. Though qualitative reports suggest that women and girls with disabilities in Nigeria experience high levels of IPV [24], there is limited evidence on their experiences.

IPV is also associated with bullying victimisation [25]. Individuals who experience IPV are likely to have a history of being bullied [26]. Like IPV, bullying is any unwanted aggressive physical or verbal behaviour rooted in power imbalance within an interpersonal relationship. It is however, perpetuated by peers who are neither siblings nor dating partners [26]. The prevalence of bullying victimisation is about the same as that of IPV [26]. Adhia et al. [26] propose that individuals who experience bullying victimisation may normalize this behaviour and then show increased tolerance for violence as they mature and enter dating relationships. This normalisation is enforced by societal norms that ignore and tolerate the bullying behaviours. Thus, patterns of maladaptive behaviour develop and evolve from routine exposure and modelling in addition to the reinforcements that follow those behaviours [26].

The aim of the present study was therefore, to determine associations between sexual identity, living with disability, HIV infection, bullying victimisation, and history of physical, emotional and sexual violence in Nigeria. It was hypothesized that sexual minorities living with a disability and HIV infection whilst experiencing bullying victimisation would be associated with physical, emotional, and sexual violence in Nigeria.

## Methods

### Ethical consideration

Approval for the conduct of this study was obtained from the Institute of Public Health Obafemi Awolowo University Research Ethics Committee (IPHOAU/12/1606). The study instrument was shared with the steering committee. The primary study was preceded by an introduction about the study team, study objectives, and time needed to complete the questionnaire. This was followed by a consent form assuring participants of the confidentiality of their responses and emphasizing that their participation was voluntary. Only participants who consented to study participation by ticking a checkbox could proceed to the survey. The survey instrument was self- administered and filled anonymously online. The questions were close-ended. The study provided a waiver of parental consent for this non-invasive online HIV and sexual and reproductive health study in line with national ethics regulation [27]. The study was carried out in line with international guidelines and guidelines of the national health research ethics committee.

### Study design

This was a secondary analysis of a primary data [28] generated through an online survey (Survey Monkey®) to seek the perspectives of respondents on the ease of access and quality of HIV prevention, treatment and ancillary care services, respect for rights, payment for services, and stigma. A secondary analysis was conducted because the study was not powered to determine the associations identified for this study. The online survey was launched on February 7, 2021 and remained open until February 19, 2021. The study recruited respondents who were 13 years and above, who were able to read, with access to the internet, and who consented to participate in the study. Restrictions were applied to the IP settings of electronic devices so that each participant could take the survey only once. Participants could edit their responses freely until they chose to submit. Email addresses were not collected to ensure anonymity.

### Sample size

The pre-survey minimum sample size for this study was set at 350 valid respondents each of the nine states, corresponding to a minimum sample size of 3150 participants. From the statistical modelling perspective, we tried to have at the national level, a minimum of 576 valid participants per State, enabling us to perform regressions with up to eight predictors with a minimum probability level (*p*-value) of 0.05.

### Study participants’ recruitment

Participants were recruited through snowballing. First, through a community consultation process led by the Coalition of Civil Society Networks on HIV and AIDS in Nigeria, nine States were identified for the survey. The nine States were spread across the six geopolitical zones in Nigeria and were the States with high prevalence of HIV in Nigeria. The selected States were Anambra and Imo States from the South East, Akwa Ibom, Delta and Rivers from the South-South, Benue from the North-Central, Kaduna from the North-West, and Lagos from the South-West, and Taraba from the North-East.

In each of the nine States, a State focal person coordinated the activities of five community representatives of the target population for the survey. The community representatives were recruited from the target populations for the survey namely: mothers (pregnant and within one-year post-partum), adolescents and young persons, and key populations (female sex workers, transgender, injecting drug users and men who have sex with other men) and members of the general population irrespective of their HIV status. Targeted recruitment of study participants from populations that were likely to use health care facilities in Nigeria helped ensure the diversity of the study population, improved the robustness of sub-group analysis [29] and improved the validity of the study findings. The State focal persons and community representatives undertook three days of online training on the study protocol, ethical considerations for conducting online surveys, and effective communication with peers.

The State focal persons conducted a pilot survey of the study tools to ensure language and cultural appropriateness. The nine State focal persons each administered the study questionnaire to 2–3 persons and identified questions that were ambiguous. The feedback was used to revise the study questionnaire by reaching a consensus with the team of State focal persons and the study leads. Next, the State community representatives piloted the revised questionnaire with 2–3 persons to ensure clarity. The finalised questionnaire when then uploaded unto SurveyMonkey® for use. The community representatives provided their peers information about the survey to establish their interest to join the research study and encourage them to participate. The links to the survey questionnaire where shared with those who showed interest, and they were encouraged to share the link with their networks. Each community representative was encouraged to recruit at least, 100 community members to take the online survey.

The survey tool was also shared with community members using Facebook, sent via WhatsApp and email and shared through other social media platforms to eligible participants. The State focal persons provided logistic and administrative support to the community representatives. They however, were not involved with study participants’ recruitment. Data collection was limited to the use of online platforms because of the COVID-19 pandemic and the need for social distancing. The use of self-completed online questionnaires, shared through social media platforms has been described as appropriate for data collection during the pandemic [30].

### Dependent variable

#### Intimate partner violence

The history of emotional violence (psychological abuse including humiliation, insults and intimidation), physical violence (the intentional use of physical force with the potential to cause injury or harm) and sexual violence (any experience of unwanted or forced sexual activity and sexual coercion) were elicited from the study participants [31]. Self-reported 12 months’ exposure to intimate partner violence was separated into three categories: (i) physical violence; (ii) sexual violence; and (iii) emotional violence. Respondents were asked to give a yes or a no response to any of these three questions. Have you been kicked, hit, slapped or otherwise physically hurt by your partner or ex-partner (physical violence)? Have you been raped or forced to have any kind of sexual activity by your partner (sexual violence)? Have you been humiliated or emotionally abused in other ways by your partner or ex-partner (emotional violence)? The three questions were adapted from the four-items HARK questionnaire each screening for past-year physical, sexual and emotional violence [32]. Each item was scored 0 (No) or 1 (Yes). The instrument had been previously used in Nigeria with a Cronbach’s alpha of 0.73 [33]. The Cronbach’s alpha in the present study was 0.80.

### Independent variables

#### Sexual identity

Information on the sexual identity (heterosexual/straight, gay, lesbian, bisexual, intersexual queer, prefer not to say) were extracted for this study analysis. Respondents were asked to identify their sexual identity by ticking a checkbox. For this study, individuals who identified themselves as being gay, lesbian, bisexual, intersexual or queer were categorized as “sexual minority”.

#### HIV status

This was assessed by a single question on self-reported HIV status with responses including opting to identify as “positive”, “negative”, “do not know” and “prefer not to report”). For the logistic regression analysis, respondent who preferred not to report their HIV status were excluded from the regression analysis. Respondents who identified that they did not know their HIV status were treated as a distinct HIV status entity because a prior study had demonstrated that men with unknown HIV status have distinct profile from that of HIV-negative and HIV-positive men [34].

#### Bullying victimisation

This was assessed with the victim subscale of the Illinois Bully Scale [35]. The subscale consists of four questions that measure both physical and verbal victimization that individuals experience from or by peers. The responses to each question ranged from never (scored 0) to 1–2 times (1), 3–4 times (2), 5–6 times (3), and 7 or more times (4). The responses were summed to derive a total score which ranged from 0 to 16. For this study, the scale was dichotomised into no (0) and yes (1–16). The scale had been validated for use in Nigeria with a Cronbach’s alpha score of 0.78 [36]. The Cronbach’s alpha in the present study was 0.91.

#### Living with disability

Respondents were asked to identify if they were living with a disability or not by checking a box (yes/no).

### Confounders

#### Sociodemographic variables

Data on age at last birthday (in years), education level completed (no formal education, primary, secondary, tertiary), sex at birth (male, female, no response) and marital status (single, married, separated/divorced or cohabiting) were collected. Cohabitation implies that a couple are living together but are not r in a civil partnership. This distinction is important for the population of sexual minority recruited in the study many of whom may cohabit rather than be married. Respondents had to check a box to indicate their sociodemographic profile.

### Data analysis

Descriptive statistics were calculated as means and standard deviations or as frequencies and percentages. T-test or chi-square were conducted to test the associations between the dependent (physical, sexual and emotional violence) and the independent and confounding variables as appropriate. Three sets of multivariate regression models were developed: one for each form of IPV. The models were adjusted for age, sex assigned at birth, marital status and education level. Odd ratios/regression coefficients and their 95% confidence intervals (CI) were calculated. Non-respondents were excluded from the bivariate and inferential statistical analysis. The IBM Statistical Package for Social Sciences, software version 23 was used for statistical analysis. Significance was set at 5%.

## Results

The 3197 respondents (92.5% of estimated sample size) had ages ranging from ranging from 13 to 72 with a mean (standard deviation) of 21.22 (15.28) years. The sample included 2374 (74.3%) sexual minorities, 117 (3.7%) persons living with disability, 620 (19.4%) HIV positive individuals and 879 (27.5%%) individuals who had experienced bullying victimisation.

As indicated in Table 1, there were significantly more heterosexual respondents (*p* < 0.001), living with disability (*p* < 0.001), with HIV positive status (*p* < 0.001), and a history of bully victimisation (*p*< 0.001) who experienced physical violence (*p* < 0.001). More respondents who were heterosexual (*p* < 0.001), living with disability (*p* < 0.001), who did not know their HIV status (*p* < 0.001), and who had a history of bully victimisation (*p* < 0.001) had experienced sexual violence (*p* < 0.001). In addition, more respondent were heterosexual (*p* < 0.001), living with disability (*p* < 0.001), HIV negative (*p* < 0.001), with a history of bully victimisation (*p* < 0.001) had experienced emotional violence (*p* < 0.001).

**Table 1:** Associations between Intimate partner violence, sexual identity, living with disability and HIV, and bully victimisation in Nigeria (*N* = 3197)

Variables	Physical violence				Sexual violence				Emotional violence				Total *N* = 3197 n (%)
	Yes *N* = 440 n (%)	No *N* = 1874 n (%)	*No response *N* = 883 n (%)	Chi-square *p*-value	Yes *N* = 341 n (%)	No *N* = 1968 n (%)	No response *N* = 888 n (%)	Chi-square *p*-value	Yes *N* = 705 n (%)	No *N* = 1631 n (%)	No response *N* = 861 n (%)	Chi-square *p*-value	
**Age**													
Mean (SD)	27.6(9.41)	29.0(10.19)		0.01	26.4(8.67)	29.1(10.24)		< 0.001	27.2(9.85)	29.3(10.21)		< 0.001	2336(73.1)
**Sexual identity**													
Heterosexual	169 (20.5)	640 (77.8)	14 (1.7)	< 0.001	106 (12.9)	697 (84.7)	20 (2.4)	< 0.001	242(29.4)	570(69.3)	11(1.3)	< 0.001	823 (25.7)
Sexual minorities	230 (17.9)	1025 (79.6)	32 (2.5)		210 (16.3)	1043 (81.0)	34 (2.6)		403(31.3)	864(67.1)	20(1.6)		1287(40.3)
*No response	41 (3.8)	209 (19.2)	837(77.0)		25 (2.3)	228 (21.0)	834(76.7)		60(5.5)	197(18.1)	830(76.4)		1087(34.0)
**Living with disability**													
Yes	33 (28.2)	81(69.2)	3(2.6)	< 0.001	27 (23.1)	88(75.2)	2(1.7)	< 0.001	50(42.7)	67(57.3)	0(0.0)	< 0.001	117 (3.7)
No	391(17.8)	1748(79.8)	52(2.4)		301 (13.7)	1832(83.6)	58(2.6)		631(28.8)	1526(69.6)	34(1.6)		2191(68.5)
*No response	16(1.8)	45(5.1)	823(93.1)		13(1.5)	48(5.4)	828(93.1)		24(2.7)	38(4.3)	827(93.0)		889(27.8)
**HIV status**													
Positive	298(17.8)	1196(71.6)	177(10.6)	< 0.001	234 (14.0)	1254(75.0)	183(11.0)	< 0.001	465(27.8)	1045(62.5)	161(9.6)	< 0.001	620 (19.4)
Negative	128 (15.1)	623(73.4)	98(11.5)		91 (10.7)	659(77.6)	99(11.7)		213(25.1)	540(63.6)	96(11.3)		1671 (52.3)
*No response	6 (1.0)	22(3.5)	592(95.5)		6 (1.0)	23(3.7)	591(95.3)		1191.8)	19(3.1)	590(95.2)		849 (26.6)
Don’t know	8 (14.0)	33(57.9)	16(28.1)		10 (17.5)	32(56.1)	15(26.3)		16(28.1)	27(47.4)	14(24.6)		57 (1.8)
**Bully Victimization**													
Yes	186(31.8)	395(67.5)	4(0.7)	< 0.001	150(25.6)	429(73.3)	6(1.0)	< 0.001	257(43.9)	326(55.7)	2(0.3)	< 0.001	585(26.6)
No	149(10.7)	1226(8.3)	14(1.0)		136(9.8)	1237(89.1)	16(1.2)		295(21.2)	1093(78.7)	1(0.1)		1389(43.5)
*No response	13(1.4)	53(5.7)	863(92.9)		6(0.6)	62(6.7)	861(92.7)		25(2.7)	47(5.1)	857(92.2)		929(29.0)
**Educational status**													
No formal education	6(14.6)	34(82.9)	1(2.4)	< 0.001	10(24.4)	37(4.3)	1(2.4)	< 0.001	12(29.3)	29(70.7)	0(0.0)	< 0.001	41(1.3)
Primary	16(15.1)	87(82.1)	3(2.8)		11 (10.4)	30(73.2)	3(2.8)		32(30.2)	73(68.9)	1(0.9)		106 (3.3)
Secondary	145(17.7)	652(79.8)	20(2.4)		91 (11.1)	92(86.8)	20(2.4)		195(23.9)	613(75.0)	9(1.1)		817 (25.6)
Tertiary	235(19.0)	969(78.4)	32(2.6)		200(16.2)	706(86.4)	38(3.1)		405(32.8)	805(65.1)	26(2.1)		1236 (38.7)
Others	28(22.0)	98(77.2)	1(0.8)		22(17.3)	998(80.7)	0(0.0)		42(33.1)	85(66.9)	0(0.0)		127(4.0)
*No response	10(1.1)	34(3.9)	826(94.9)		7(0.8)	105(82.7)	826(94.9)		19(2.2)	26(3.0)	825(94.8)		870(27.1)
**Marital status**													
Single	273(20.5)	1021(76.7)	38(2.9)	< 0.001	230(17.3)	1061(79.7)	41(3.1)	< 0.001	444(33.3)	858(64.4)	30(2.3)	< 0.001	1332 (41.7)
Married	97 (12.6)	658(85.5)	15(1.9)		57 (7.4)	699(90.8)	14(1.8)		152(19.7)	615(79.9)	3(0.4)		770 (24.1)
Divorced/Separated	38 (27.5)	98(71.0)	2(1.4)		27 (19.6)	107(77.5)	4(2.9)		57(41.3)	79(57.2)	2(1.4)		138 (4.3)
Cohabiting	25 (29.1)	61(70.9)	0(0.0)		22 (25.6)	63(73.3)	1(1.2)		35(40.7)	51(59.3)	0(0.0)		86 (3.7)
*No response	7(0.8)	36(4.1)	828(95.1)		5(0.6)	38(4/4)	828(95.1)		17(2.0)	28(3.2)	826(94.8)		871(27.1)
**Sex at birth**													
Male	177 (16.5)	869(81.1)	26(2.4)	< 0.001	138 (12.9)	901(84.0)	33(3.1)	< 0.001	331(30.9)	720(67.2)	21(2.0)	< 0.001	1072 (33.5)
Female	256 (20.4)	973(77.4)	28(2.2)		199 (15.8)	1032(82.1)	26(2.1)		358(28.5)	887(70.6)	358(28.5)		1257 (39.3)
*No response	7 (0.8)	32(3.7)	829(95.5)		4 (0.5)	35(4.0)	829(95.5)		16(1.8)	24(2.8)	828(95.4)		868 (27.2)

^*^ Excluded from the statistical analysis

Respondents reporting physical, sexual and emotional violence were significantly older (*p* < 0.001); divorced/separated or cohabiting (*p* < 0.001). In addition, more women experienced physical (*p* < 0.001) and sexual (*p* < 0.001) violence while more men experienced emotional violence (*p* < 0.001). Finally, respondents with no formal education are less likely to experience IPV (*p* < 0.001).

Table 2 shows that respondents who identify as HIV positive (AOR: 2.01; 95% CI: 1.46–2.76; *p* < 0.001); who had a history of bullying victimisation (AOR: 3.79; 95% CI: 2.86 – 5.68; *p* < 0.001), and who were females (AOR: 1.52; 95% CI: 1.13–2.043; *p* < 0.001) had significantly higher odds of physical violence when compared with respondents who were HIV negative had no history of bullying victimisation and who were males respectively. Respondents who were married have significantly lower odds of experiencing physical violence compared to respondents who were single (AOR: 0.66; 95% CI: 0.45 – 0.96; *p* = 0.03).

**Table 2:** Binary logistic regression analysis of the associations between intimate partner violence and sexual identity, living with disability and HIV, and bully victimisation in Nigeria (*N*=3197)

Variables	Ever experienced Intimate partner violence					
	Physical violence AOR (95%CI)	*p*-value	Sexual violence AOR (95%CI)	*p*-value	Emotional violence	*p*-value
**Sexual identity**						
Heterosexual	1.00	-	1.00	-	1.00	-
Sexual minorities	0.93 (0.69–1.23)	0.600	1.20 (0.88–1.64)	0.258	1.04 (0.82–1.32)	0.751
**Living with disability**						
No	1.00	-	1.00	-	1.00	-
Yes	1.38 (0.79–2.41)	0.253	1.49 (0.83–2.65)	0.179	1.54 (0.95–2.49)	0.081
**HIV status**						
Negative	1.00	-	1.00	-	1.00	-
Positive	2.01 (1.46–2.76)	< 0.001	2.17 (1.55–3.05)	< 0.001	1.59 (1.24–2.06)	< 0.001
Don’t know	2.33 (0.96–5.68)	0.062	3.03 (1.20–7.62)	0.019	1.45 (0.65–3.20)	0.361
**Bullying victimisation**						
No	1.00	-	1.00	-	1.00	-
Yes	3.79 (2.86–5.68)	< 0.001	3.05 (2.27–4.10)	< 0.001	2.66 (2.10–3.37)	< 0.001
**Age**	0.99 (0.97–1.01)	0.312	0.97 (0.95–0.99)	0.016	0.99 (0.97–1.00)	0.047
**Educational status**						
No formal education	1.00	-	1.00	-	1.00	-
Primary	1.35 (0.33–5.61)	0.676	0.73 (0.19–2.84)	0.644	1.05 (0.37–2.98)	0.935
Secondary	1.47 (0.41–5.28)	0.556	0.65 (0.20–2.09)	0.464	0.61 (0.24–1.55)	0.296
Tertiary	1.66 (0.47–5.95)	0.435	1.16 (0.37–3.72)	0.798	1.12 (0.44–2.83)	0.816
Others	2.40 (0.61–9.44)	0.212	1.45 (0.41–5.31)	0.548	1.24 (0.45–3.46)	0.676
**Marital status**						
Single	1.00	-	1.00	-	1.00	-
Married	0.66 (0.45–0.96)	0.029	0.40 (0.26–0.62)	< 0.001	0.68 (0.50–0.93)	0.015
Divorce/Separated	1.67 (0.96–2.90)	0.070	1.43 (0.79–2.59)	0.232	2.05 (1.25–3.35)	< 0.001
Cohabiting	1.75 (0.98–3.11)	0.059	1.53 (0.84–2.79)	0.163	1.95 (1.12–3.28)	0.012
**Sex at birth**						
Male	1.00	-	1.00	-	1.00	-
Female	1.52 (1.13–2.03)	< 0.001	1.83 (1.34–2.50)	< 0.001	1.02 (0.80–1.30)	0.866

Also, respondents who identify as HIV positive (AOR: 2.17; 95% CI: 1.55–3.05; *p* < 0.001), who don’t know their HIV status (AOR: 3.03; 95%CI: 1.20–7.62; *p* = 0.019), who had a history of bullying victimisation (AOR: 3.05; 95% CI: 2.27 – 4.10; *p* < 0.001) and who were females (AOR: 1.83; 95% CI: 1.34 – 2.50; *p* < 0.001) had higher odds of sexual violence when compared with respondents who were HIV negative, had no history of bullying victimisation and who were males respectively. Older respondents had significantly higher odds of experiencing sexual violence (AOR: 0.97; 95% CI: 0.95–0.99; *p* = 0.016). Respondents who were married have significantly lower odds of experiencing sexual violence compared to respondents who were single (AOR: 0.40; 95% CI: 0.26 – 0.62; *p* < 0.001).

In addition, respondents who identify as HIV positive (AOR: 1.59; 95% CI: 1.24–2.06; *p* < 0.001), who had a history of bullying victimisation (AOR: 2.66; 95% CI: 2.10 – 3.37; *p* < 0.001), who were divorced/separated (AOR: 2.05; 95% CI: 1.25 – 3.35; *p* < 0.001) or cohabiting violence (AOR: 1.95; 95% CI: 1.12 – 3.28; *p* = 0.012) had higher odds for emotional violence when compared with respondents who were HIV negative, had no history of bullying victimisation, and who were single respectively. Respondents who were married have significantly lower odds of experiencing emotional violence when compared with singles (AOR: 0.68; 95% CI: 0.50 – 0.93; *p* = 0.015).

## Discussion

The present study findings suggests that that HIV positive individuals and those with a history of bullying victimisation were more likely to experience physical, sexual and emotional violence. Also, those living with a disability and sexual minority individuals have no significant difference in their risk for IPV when compared with the individuals who were not living with disability and who are heterosexuals.

This study has a number of strengths. First, the large sample size offers the opportunity for robust sub-group analysis and thereby, enabling this study provide new information about IPV among sexual minority individuals in Africa. It is also one of the few studies to explore experiences of IPV among sexual minority individuals and people living with disability in Nigeria. Second, the study applied standardised instruments to measure the constructs under investigation thereby offering further validation of the study findings.

The study however, has some limitations with implications for cautious interpretation of the study findings. First, this is a cross sectional, study and a cause effect relationship cannot be confirmed. Second, a convenient sample with over-representative of sexual minority individuals. This prevents generalizability of study findings beyond the population studied. Third, the online data collection process likely excluded participants with no smartphones or internet access. These are likely to be persons with lower education and income status. This limits the generalisability of the study findings to all socioeconomic groups in Nigeria. Fourth, this is a secondary data analysis that comes with the inherent problem of analytical errors [37]. Finally, this self-reported data may be subject to misreporting of self-assessed measures of phenomena like HIV positivity status [38] and experience of IPV [39]; and there is a prospect for bias reporting with the measurement of bullying victimisation [40]. The use of validated instruments however, helped ensure the responses provided measured the construct of interest while we are unable to reduce the risk for false reporting due to social desirability bias.

A worthwhile consideration observed here was the significant association found between three types of IPV, bullying victimisation, and HIV positive status. Prior research has indicated that relationships exist between bullying and being a victim of IPV [26, 41–44], and between IPV and HIV positive status [45–50]. Bullying victimisation is also associated with increased risk for HIV infection [51, 52]. Adhia et al. [26] suggested that the experience of bullying victimization can lead to the development of personality and behavioral trait nuances resulting in individuals becoming socially withdrawn. This impact of bullying may increase vulnerability, exclusion, future IPV and thereby, may increase risk of HIV infection. Future studies may explore the association between bullying victimisation and the risk for HIV infection; and develop a theoretical framework that defines the link paths between IPV, bullying victimisation, and HIV status. These links may likely be complex.

A pointer to this complexity is the observed association between unknown HIV status and increased risk for sexual violence. Prior studies had demonstrated that sexual violence is associated with increased risk for HIV infection [53] and that gender-based violence impedes uptake of HIV testing [54] due to the fear of a violent reaction from one’s partner in the event of a positive test [55, 56]. These prior studies had associated physical violence with these highlighted risk [57–59] while the current study suggests that sexual violence may also reduce the likelihood of getting tested for HIV infection. This postulation needs to be further investigated.

Bullying victimisation and IPV have a negative impact on the mental and physical health of affected individuals. For Nigeria facing an epidemic of bullying victimisation, IPV and HIV infection—the experience of bullying in schools’ ranges from 33 to 85% [60]; the experience of IPV ranges from 29%—79% [61], and Nigeria has the second highest rate of new HIV and highest burden of HIV in the world [62] – these findings may suggest that the country may be predisposed to a huge crisis of future of mental health problems. This crisis may be worse for females who, in the light of the finding of our study, may be more at risk of IPV and who are more at risk of experiencing mental health problems [63]. Our study findings suggest that there is the potential for a vicious cycle of bullying victimisations, HIV infection and IPV to emerge. This makes it critical for the country to institute programs that can mitigate the risk for bullying victimisations, HIV infection and IPV.

A prior report from the community of women living with disability in Nigeria indicated that women with disability are twice as likely to experience gender-based violence (IPV is a form of gender-based violence [64]) than other women [65]. Disability related social stigma triggers effects for the perpetuation of emotional violence [66] and the vulnerability of people living with disability to IPV increases with the severity of the disability [67]. We found no evidence to suggest that people living with a disability had significant higher odds of experiencing any of the three forms of IPV that we studied when compared with respondents who do not live with disability. The data however, did indicate that people with disability may be at higher risk for all three forms of IPV even if the findings were not significant. More studies are required to understand the findings of this study, and to explore how the culture context may moderate the associations between disability and IPV.

Also, sexual minority individuals may have a high risk of experiencing IPV [68–70] due to the stress resulting from stigma, which then breeds or exacerbates partner violence [71] or from partner jealousy [69]. We found that the risk of IPV by sexual minority individuals did not significantly differ from that of heterosexuals. This present study is the first report the experience of IPV by sexual minority individuals in Nigeria. In Nigeria, many sexual minority individuals fail to disclose their sexual or gender identity because of the harsh political, legal, cultural, and religious environment [72]. While non-disclosure of sexual identity may protect sexual minority individuals from IPV, it may cause undue mental distress and a negative impact on physical health and psychological wellbeing, which can be similar to the ill-effects of IPV [73]. It may be important to conduct studies exploring how non-disclosures of sexual identify by sexual minority individuals may increase the risk for poor physical and psychological health. It is also possible that an awareness of the risk for minority stress may potentially increase a sense of responsibility of sexual minority sexual partners to one another thereby limiting to the experience of IPV among co-habiting sexual minority individuals. This postulation also needs to be explored through future studies.

Finally, this study highlighted that individuals who were divorced or separated have significantly higher odds of experiencing emotional violence when compared with singles. Also, females have a higher risk of experiencing physical and sexual violence and younger participants have a higher risk of experiencing sexual violence. Past research has indicated that females [74] and younger individuals [75] are at significantly increased risk for IPV. The present findings provide additional evidence to support prior findings, suggests the universality of the experience of IPV [76], and also the commonalities in the risk factors for IPV in different cultures. IPV is an expression of relational power, dominance and control and thus, where there is the endorsement of traditional masculinity ideology, the risk for IPV is high where such dominance can be expressed like in male/female relationships and younger/older person relationships [77]. However, unlike past studies that indicated that formal/co-habiting relationships [78] increases the risk for IPV as marital relationships is a place where dominance can be expressed in communities with traditional masculinity ideology like Nigeria, we found that marital relationships seem to be protective from IPV. Still ironical is that those who cohabit have higher odds of experiencing, emotional violence than singles. It is not clear why cohabiting may increase the risk for emotional violence and being married reduces the risk for same; or why divorcee and those who had separated will experience more emotional violence than those who are married. These study findings suggest that there is a complex relationship between IPV and marital status and further studies are needed to explore the study findings.

As society change, the rebuttal of traditional masculinity ideology increases thereby reducing the risk for continuing in relationships that predisposes to violence. Thus, more people may embrace being divorced/separated to reduce their risk of trauma from IPV. This postulation may inform the observed higher odds of a history of IPV among those that are divorced/separated and lower odds of IPV observed among those who continue in a marital relationship. The results should be taken with caution as there is a risk that those who are in marital relationships may under-report their experience of IPV while those who are divorced/separated may have exaggerated perceptions of their experiences including the experience of IPV. All the same, societies change with time and so do demographics related to marriage [79, 80]. These changes in global demographics may reflect a potential for the diminution of the experience of IPV and bullying victimisation in the future. Nevertheless, in the current space of time, actions need to be taken to further reduce the risk of populations vulnerable to all forms of IPV.

In conclusion, there are complex relationships between different types of IPV, sexual identity, HIV infection status, disability status and bullying victimisation. Age, sex at birth, marital status, and educational status may moderate these relationships taking cognisance of the cultural context in which these complex relationships occur. Future relational analysis is necessary to further unravel the direction of pathways between these variables.

## Supplementary Information

**Additional file 1.** 

**Additional file 2:** 

## Data Availability

All data generated for this study are presented in the manuscript. The raw data that support the findings of this study are available from Morenike Oluwatoyin Folayan but restrictions apply to the availability of these data, which were used under license for the current study, and so are not publicly available. Data are however available from the authors upon reasonable request and with permission of Morenike Oluwatoyin Folayan.

## Abbreviations

AOR: Adjusted Odds Ratio
CI: Confidence interval
HIV: Human immunodeficiency virus
IPV: Intimate Partner Violence
