# Supplementary material for: Associations between sexual identity, living with disability, bully victimisation, and HIV status and intimate partner violence among residents in Nigeria
Source: BMC Public Health. 2022 Sep 16;22:1756. doi: 10.1186/s12889-022-14186-6 (PMC9479364; doi:10.1186/s12889-022-14186-6)
Supplement: Supplementary file 1 — Additional file 1. [file 12889_2022_14186_MOESM1_ESM.pdf]

HEALTH RESEARCH ETHIC COMMITTEE (HREC)
INSTITUTE OF PUBLIC HEALTH
OBAFEMI AWOLOWO UNIVERSITY, ILE-IFE, NIGERIA.

Our Ref: HREC NO: IPH/OAU/12/1606

Your Ref: ______________

Date: February 8th, 2021

Notice of Full Approval after Full Committee Review

COMMUNITY LED MONITORING ON QUALITY OF HIV SERVICE DELIVERY

Health Research Ethics Committee assigned number: IPHOAU/12/1606

Applicant's Name: PROF. MORENIKE OLUWATOYIN UKPONG, IKENNA NWAKANMA

Applicant's Address: Dept. of Child Dental Health, Faculty of Dentistry, OAU, Ile-Ife.

Date of receipt of valid application: 4th February, 2021

Date of meeting when final determination of research was made: February 5th, 2021

This is to inform you that the research described in the submitted protocol (HREC No: IPHOAU/12/1606), the consent forms and other participant information materials have been reviewed and *given full approval by the Health Research Ethics Committee.* This approval dates from Feb. 5th, 2021 to Feb. 4th, 2022. If there is delay in starting the research, please inform the HREC so that the dates of approval can be adjusted accordingly. Note that no participant accrual or activity related to this research may be conducted outside of these dates. *All informed consent forms used in this study must carry the HREC assigned number and duration of HREC approval of the study.* In multiyear research, endeavor to submit your annual report to the HREC early in order to obtain renewal of your approval to avoid disruption of your research.

The National Code for Health Research Ethics requires you to comply with all institutional guidelines, rules and regulations and with the tenets of the Code including ensuring that all adverse events are reported promptly to the HREC. No changes are permitted in the research without prior approval by the HREC except in circumstances outlined in the Code. The HREC reserves the right to conduct compliance visit to your research site without previous notification.

HEALTH RESEARCH
ETHICS COMMITTEE.
O.A.U, ILE-IFE

------SIGN: [signature] DATE: 8/2/2021

Chairman, HREC

Web-site: www.iphoau.org // E-mail: iph@oauife.edu.ng, iphhoauife@gmail.com

CamScanner
