# Supplementary material for: Associations between sexual identity, living with disability, bully victimisation, and HIV status and intimate partner violence among residents in Nigeria
Source: BMC Public Health. 2022 Sep 16;22:1756. doi: 10.1186/s12889-022-14186-6 (PMC9479364; doi:10.1186/s12889-022-14186-6)
Supplement: Supplementary file 2 — Additional file 2: [file 12889_2022_14186_MOESM2_ESM.pdf]

COMMUNITY ASSESSMENT OF THE QUALITY OF SERVICES DELIVERY FOR PLHIV, ADOLESCENTS AND KEY POPULATIONS IN NIGERIA

Informed Consent Document

The objective of this survey is to identify the gaps in the quality of HIV prevention and treatment service delivery for people living with HIV, adolescents and key populations in Nigeria. The aim is to be able to make evidence-based recommendation to government and donor interventions to address the needs of community members in Nigeria.

THE SURVEY WILL TAKE ABOUT 10 MINUTES OF YOUR TIME.

The survey data is being collected anonymously, it is unlinked and there will be no personal data collected. This survey will not cause you social, economic, legal, physical or financial risk that is more than those experienced in your routine day to day life. Please note that all data generated from this study shall be kept confidential. The stored data shall not include any form of identification of the respondents. Only the consultant and her research team members can have access to the data.

When the survey is concluded and the data analyzed, the summary findings will be validated and shared with the government and the donor agencies. Community representatives will be able to use the outcome of this survey to inform the PEPFAR COP21 plan development process and other national government ongoing planning process to address the HIV prevention and care needs of Nigerians. The findings should also feed into the national HIV and AIDS strategic plan mid-term review process.

You are free not to respond to questions you may not want to respond too. Please do give sincere and honest answers.

* 1. Please check the box below. You will not be able to proceed to answer the questions if all three boxes are not checked.

- ☐ I have read and understood the details provided above about this study.
- ☐ I consent to participate in the study.
- ☐ I do not consent to participate in the study.

COMMUNITY ASSESSMENT OF THE QUALITY OF SERVICES DELIVERY FOR PLHIV, ADOLESCENTS AND KEY POPULATIONS IN NIGERIA

Section 1.0
Access to HIV prevention services

2. Where do you access HIV prevention services?

- [ ] Public (government owned) hospital
- [ ] Private for profit hospital
- [ ] Private not for profit hospital
- [ ] One stop shops
- [ ] Faith based organizations
- [ ] I have never accessed HIV prevention services (Skip to Section 2.0)

Others (please mention)

____

COMMUNITY ASSESSMENT OF THE QUALITY OF SERVICES DELIVERY FOR PLHIV, ADOLESCENTS AND KEY POPULATIONS IN NIGERIA

Section 1.0
Access to HIV prevention services

3. Which of these HIV prevention services will you like to access (tick as many options)?

- ☐ HIV testing services
- ☐ HIV self-testing services
- ☐ Male condoms
- ☐ Female condoms
- ☐ Pre-exposure prophylaxis
- ☐ Post exposure prophylaxis
- ☐ Voluntary male circumcision

4. Which of these HIV prevention service were provided at the health facility you attended(tick as many options)?

- ☐ HIV testing services
- ☐ HIV self-testing services
- ☐ Male condoms
- ☐ Female condoms
- ☐ Pre-exposure prophylaxis
- ☐ Post exposure prophylaxis
- ☐ Voluntary male circumcision

5. How easy was it for you to access any of these services?

- ○ Very easy
- ○ Easy
- ○ Neutral
- ○ Difficult
- ○ Very difficult

6. How satisfied were you with the quality of the service received?

- ○ Very Satisfied
- ○ Satisfied
- ○ Neutral
- ○ Dissatisfied
- ○ Very dissatisfied

7. Who funds the operations of the facility?

- ○ Nigeria government
- ○ Private
- ○ PEPFAR
- ○ Global Fund
- ○ Faith missions
- ○ Don’t know

COMMUNITY ASSESSMENT OF THE QUALITY OF SERVICES DELIVERY FOR PLHIV, ADOLESCENTS AND KEY POPULATIONS IN NIGERIA

Section 2.0
Access to ancillary care services

8. Did you receive counselling on sexual and reproductive health at the facility?

- ◯ Yes
- ◯ No
- ◯ Don’t know

9. Which of these sexual and reproductive health counselling did you receive?

- ☐ How one should disclose their HIV status to a sexual partner
- ☐ Different condom options
- ☐ How to use condoms during sex for HIV/STD and pregnancy prevention
- ☐ Choices and use of modern contraceptives (male/female condoms, IUDs, implants, injectable contraceptives, pills, tubal ligation/vasectomy)
- ☐ Regular STI screening and treatment

Other (please specify)

10. What other ancillary care would you have loved to receive (tick as many options)?

- ☐ HIV self-testing
- ☐ Mental health
- ☐ Tuberculosis
- ☐ Malaria
- ☐ Anal cancer screening
- ☐ Prostate cancer screening
- ☐ Cervical cancer screening
- ☐ Hypertension management
- ☐ Diabetes management
- ☐ Viral hepatitis management
- ☐ Gender based violence management

Other (please specify)

11. Which of these ancillary care services did you receive (tick as many options)?

- [ ] HIV self-testing
- [ ] Mental health
- [ ] Tuberculosis
- [ ] Malaria
- [ ] Anal cancer screening
- [ ] Prostate cancer screening
- [ ] Cervical cancer screening
- [ ] Hypertension management
- [ ] Diabetes management
- [ ] Viral hepatitis management
- [ ] Gender based violence management

Other (please specify)

12. Have you ever been screened for any of the following in a health facility (tick as many options)?

- [ ] Mental health
- [ ] STI risk assessment
- [ ] Syphilis
- [ ] Gonorrhea
- [ ] Chlamydia
- [ ] HPV/Genital warts
- [ ] Genital herpes
- [ ] HIV

13. Will you like to be screened for any of the following in a health facility (tick as many options)?

- [ ] Mental health
- [ ] STI risk assessment
- [ ] Syphilis
- [ ] Gonorrhea
- [ ] Chlamydia
- [ ] HPV/Genital warts
- [ ] Genital herpes
- [ ] HIV

14. How easy was it for you to access any of these services?

- Very easy
- Easy
- Neutral
- Difficult
- Very difficult

15. How satisfied were you with the quality of the service received?

- Very Satisfied
- Satisfied
- Neutral
- Dissatisfied
- Very dissatisfied

16. Who funds the operations of the facility?

- Nigeria government
- Private facility
- Faith based organization
- PEPFAR
- Global Fund
- Don’t know

COMMUNITY ASSESSMENT OF THE QUALITY OF SERVICES DELIVERY FOR PLHIV, ADOLESCENTS AND KEY POPULATIONS IN NIGERIA

Section 3.0
Section for people living with HIV

17. Are you living with HIV?

- ◯ Yes
- ◯ No (Skip to Section 4.0)
- ◯ Don’t know

COMMUNITY ASSESSMENT OF THE QUALITY OF SERVICES DELIVERY FOR PLHIV, ADOLESCENTS AND KEY POPULATIONS IN NIGERIA

Section 3.0

Section for people living with HIV

18. How long have you been living with HIV?

19. How long after you got diagnosed with HIV did you receive ARVs?

20. How is antiretroviral services offered in the facility you attend?

- No separation of Pediatric, Adolescent, and Adult (general clinic)
- Pediatric separate from Adolescent & Adult
- Pediatric and Adolescent separate from Adult
- Pediatric, Adolescent, and Adult all separate

21. Is multi-month scripting for people living with HIV available at the health facility?

- Yes
- No
- Don’t know

22. If yes, which multi-month scripting schedule is done in your facility? Select all that apply.

- 2 months
- 3 months
- 4 months
- 6 months
- Not available
- Don’t know

Other (please specify)

23. Is community pharmacy services available to you as a PLHIV at the facility?

- ◯ Yes
- ◯ No
- ◯ Don’t know

24. If yes, how satisfied are you with this service?

- ◯ Very Satisfied
- ◯ Satisfied
- ◯ Neutral
- ◯ Dissatisfied
- ◯ Very dissatisfied

25. Which of these services do you receive free of charge (tick all that applies)?

- ☐ HIV testing
- ☐ Antiretroviral therapy
- ☐ Viral load
- ☐ CD4 count
- ☐ Pregnancy tests
- ☐ Condoms
- ☐ Contraception: Oral pills
- ☐ Contraception: injectables
- ☐ Contraception: IUCD
- ☐ Contraception: implant
- ☐ Contraception: Sterilisation
- ☐ Mental health screening
- ☐ STI risk assessment
- ☐ Syphilis treatment
- ☐ Gonorrhea treatment
- ☐ Chlamydia treatment
- ☐ HPV/Genital warts treatment
- ☐ Genital herpes treatment
- ☐ Tuberculosis screening
- ☐ Tuberculosis treatment
- ☐ Malaria treatment
- ☐ Mosquito nets

26. Which of these services do you pay for and how much?

HIV testing	
Antiretroviral therapy	
Viral load	
CD4 count	
Pregnancy tests	
Condoms	
Contraception: Oral pills	
Contraception: injectables	
Contraception: IUCD	
Contraception: implant	
Contraception: Sterilisation	
Mental health screening	
STI risk assessment	
Syphilis treatment	
Gonorrhea treatment	
Chlamydia treatment	
HPV/Genital warts treatment	
Genital herpes treatment	
Tuberculosis screening	
Tuberculosis treatment	
Malaria treatment	
Mosquito nets	

27. Overall, how easy was it for you to access any of these services?

- Very easy
- Easy
- Neutral
- Difficult
- Very difficult

28. Overall, how satisfied were you with the quality of the service received?

- ◯ Very Satisfied
- ◯ Satisfied
- ◯ Neutral
- ◯ Dissatisfied
- ◯ Very dissatisfied

29. Who funds the operations of the facility?

- ◯ Nigeria government
- ◯ Private
- ◯ PEPFAR
- ◯ Global Fund
- ◯ Faith based organisation
- ◯ Don’t know

COMMUNITY ASSESSMENT OF THE QUALITY OF SERVICES DELIVERY FOR PLHIV, ADOLESCENTS AND KEY POPULATIONS IN NIGERIA

Section 4.0
HIV Health Care Service Delivery

30. How satisfied were you with the attitude or delivery of HIV services your the last service delivery point?

- ◯ Very satisfied
- ◯ Satisfied
- ◯ Neutral
- ◯ Dissatisfied
- ◯ Very dissatisfied

31. Were services respectfully delivered?

- ◯ Yes
- ◯ Neutral
- ◯ No

32. How comfortable where those service delivery points?

- ◯ Very comfortable
- ◯ Comfortable
- ◯ Neutral
- ◯ Uncomfortable
- ◯ Very Uncomfortable

33. Where service delivered on time?

- ◯ Yes
- ◯ Neutral
- ◯ No

34. How organized were the service providers?

- ○ Very organized
- ○ Organized
- ○ Neutral
- ○ Unorganized
- ○ Very unorganized

35. Have you ever been stigmatized at the health care facility?

- ○ Yes
- ○ Neutral
- ○ No

36. Do you plan to continue to receive services at the health facility where you are currently receiving services?

- ○ Yes
- ○ Neutral
- ○ No

COMMUNITY ASSESSMENT OF THE QUALITY OF SERVICES DELIVERY FOR PLHIV, ADOLESCENTS AND KEY POPULATIONS IN NIGERIA

Section 5.0
Respect for human rights

37. Has any of this your rights been violated during receipt of HIV prevention and treatment services in any facility in Nigeria

	Yes	No	Don't know
Right to relevant information in a language and manner the patient understands	◯	◯	◯
Right to timely access to detailed and accurate medical records and available services	◯	◯	◯
Right to transparent billing and full disclosure of any costs, including recommended treatment plans	◯	◯	◯
Right to privacy, and confidentiality of medical records	◯	◯	◯
Right to clean, safe, and secure healthcare environments	◯	◯	◯
Right to be treated with respect, regardless of gender, race, religion, ethnicity, allegations of crime, disability or economic circumstances	◯	◯	◯
Right to receive urgent, immediate and sufficient intervention and care, in the event of an emergency	◯	◯	◯
Right to reasonable visitation in accordance with prevailing rules and regulations	◯	◯	◯

	Yes	No	Don't know
Right to decline care, subject to prevailing laws and upon full disclosure of the consequences of such a decision	◯	◯	◯
Right to decline or consent to participation in medical research, experimental procedures or clinical trials	◯	◯	◯
Right to quality care in accordance to prevailing standards	◯	◯	◯
Right to complain and express dissatisfaction regarding services received	◯	◯	◯

COMMUNITY ASSESSMENT OF THE QUALITY OF SERVICES DELIVERY FOR PLHIV, ADOLESCENTS AND KEY POPULATIONS IN NIGERIA

Section 6
Intimate partner violence and bully victimisation

38. Has any of this your rights been violated during receipt of HIV prevention and treatment services in any facility in Nigeria

	Yes	No
Have you been humiliated or emotionally abused in other ways by your partner or ex-partner?	◯	◯
Have you been afraid of your partner or ex-partner?	◯	◯
Have you been raped or forced to have any kind of sexual activity by your partner?	◯	◯
Have you been kicked, hit, slapped or otherwise physically hurt by your partner or ex-partner?	◯	◯

39. For each of the following questions, choose how many times these things happened to you as a child or as an adolescent

	Never	1-2 times	3-4 times	5-7 times	7 or more times
Other children picked on me	◯	◯	◯	◯	◯
Other children made fun of me	◯	◯	◯	◯	◯
Other children called me names	◯	◯	◯	◯	◯
I got hit and pushed by other children	◯	◯	◯	◯	◯

COMMUNITY ASSESSMENT OF THE QUALITY OF SERVICES DELIVERY FOR PLHIV, ADOLESCENTS AND KEY POPULATIONS IN NIGERIA

Section 7.0
Biodata

40. Sex at birth

- ◯ Male
- ◯ Female

41. Gender identity

- ◯ Male
- ◯ Female
- ◯ Transgender
- ◯ Others

42. Sexual orientation

- ◯ Heterosexual
- ◯ Lesbian
- ◯ Gay
- ◯ Bisexual
- ◯ Intersexual
- ◯ Queer
- ◯ Others

43. Age

[ ]

44. Educational level

- ◯ None
- ◯ Primary
- ◯ Secondary
- ◯ Tertiary
- ◯ Others

45. Marital Status

- ◯ Single
- ◯ Married
- ◯ Separated
- ◯ Divorced
- ◯ Cohabitation

46. Are you living with disability

- ◯ No
- ◯ Yes
- ◯ No response

47. Has any disability you have limited your access to HIV services?

- ◯ No
- ◯ Yes
- ◯ Don’t know
